# Molecular identification and antifungal susceptibility profile of *Aspergillus flavus* isolates recovered from clinical specimens in Kuwait

**DOI:** 10.1186/1471-2334-13-126

**Published:** 2013-03-06

**Authors:** Faten Al-Wathiqi, Suhail Ahmad, Ziauddin Khan

**Affiliations:** 1 Department of Microbiology, Faculty of Medicine, Kuwait University, P. O. Box 24923, 13110, Safat, Kuwait

**Keywords:** *Aspergillus flavus*, Molecular characterization, β-tubulin and calmodulin genes, Antifungal susceptibility, Etest, Triazoles, Echinocandins, Amphotericin B

## Abstract

**Background:**

Within the genus *Aspergillus*, *A. flavus* is the second most important species of clinical significance. It is predominantly associated with infections involving sinuses, eye and skin, mostly in geographic regions with hot and arid climate, including the Middle East. Recent reports on emergence of resistance to triazoles among *Aspergillus* spp. is a cause of concern for treatment of patients with invasive aspergillosis. In this study we present data on genetic characterization and antifungal susceptibility profile of clinical and environmental isolates of *A. flavus.*

**Methods:**

Ninety-nine *Aspergillus* section *Flavi* isolates, originating from clinical (n=92) and environmental (n=7) sources, initially identified by morphological characteristics, were analyzed by partial sequencing of β-tubulin and calmodulin gene fragments and their susceptibilities to six antifungal agents was determined by Etest on RPMI1640 and Muller-Hinton agar media. Etest minimum inhibitory concentrations (MICs) of amphotericin B and voriconazole were also compared with zone of inhibition diameters obtained by disc diffusion test on RPMI agar medium.

**Results:**

The identity of all clinical and environmental isolates was confirmed as *A. flavus* species by combined analysis of β-tubulin and calmodulin genes. The mean MIC_90_ (μg/ml) values on RPMI medium for amphotericin B, voriconazole, posaconazole, anidulafungin, micafungin and caspofungin were 3, 0.25, 0.25, 0.002, 0.002 and 0.032, respectively. No environmental isolate exhibited MIC value of >2 μg/ml for amphotericin B. For clinical isolates, the zone of inhibition diameters for amphotericin B and voriconazole ranged from 7–16 mm and 24–34 mm, respectively. Linear regression analysis between Etest MIC values and disk diffusion diameters revealed a significant inverse correlation with amphotericin B (*p* <0.001) and voriconazole (*p*<0.003).

**Conclusions:**

The β-tubulin and calmodulin gene sequences confirmed that all 92 clinical isolates identified phenotypically belonged to *A. flavus* taxon*,* thus suggesting that the other species within *Aspergillus* section *Flavi* are of little clinical significance. Triazoles and echinocandins showed very good *in vitro* activity against the *A. flavus,* however, 10% clinical isolates showed MICs of >2 μg/ml for amphotericin B.

## Background

Among filamentous fungal pathogens, *Aspergillus* spp. account for highest rates of morbidity and mortality among severely immunocomnpromised patients [1]. Although *A. fumigatus* is the principal etiologic agent of invasive aspergillosis, the etiologic role of non-*fumigatus Aspergillus* species is being increasingly recognized [2-4]. *Aspergillus flavus* is the second most important species causing localized as well as systemic infections [2,5-7]. The species is of particular significance in North Africa, India and the Middle East, where it is predominantly associated with nasal/sinus infections [8-12]. In 2008, Clinical and Laboratory Standards Institute produced standard guidelines based on broth microdilution (BMD) method for determining antifungal susceptibilities of spore-forming filamentous fungi [13]. Recently, epidemiologic cutoff values (ECVs) for triazoles and caspofungin for wild-type strains of *Aspergillus* spp. have been developed [14-16]. However, due to interspecies/inter-strain differences in MICs, clinical breakpoints for each *Aspergillus* spp. are not yet available. Occurrence of azole resistance among wild-type strains of *A. fumigatus* due to mutations in *cyp51A* gene and possibility of finding similar resistance in other *Aspergillus* spp [17,18] have necessitated the need to evaluate efficacy of simple agar-based methods for antifungal susceptibility testing. Preliminary studies have suggested that disk diffusion test and Etest show comparable results with BMD method for susceptibility testing with azoles [19-22]. Here, we present molecular characterization and MIC results of six antifungal agents tested against 99 *A. flavus* isolates by Etest and compare amphotericin B and voriconazole MICs with zone of inhibition diameters obtained by disk diffusion test.

## Methods

### *Aspergillus flavus* isolates

Ninety-two clinical and 7 environmental isolates of *A. flavus* were included in the study (Table 1). The clinical isolates were recovered from a variety of specimens over an 18-year period (1993–2011). They were deposited in the culture collection of the Mycology Reference Laboratory and maintained by periodic sub-culture. Since the isolates were obtained during routine mycological investigations, there was no ethical requirement to take approval from individual patients for their subsequent use. The study (Project No. YM 03/10) was approved by the Ethical Committee of Faculty of Medicine, Health Sciences Center, Kuwait University. All isolates were identified as *A. flavus* by typical colony and microscopic characteristics as described by Klich [23]. The isolates were also characterized and speciated by molecular methods.

**Table 1:** **Sources of *Aspergillus flavus *isolates used for *in vitro *antifungal susceptibility by Etest**

**Sources**	**No. of isolates**
Ear swabs	31
Nasal biobsy	10
Respiratory secretion*	32
Wound swabs	9
Rectal swab	2
Peritoneal abscess	3
Blood	1
Corneal plate	1
Urine	1
Cutaneous infections	2
Environment	7
Total	99

*Include sputum, endotracheal secretion and bronchoalveolar lavage.

### Molecular characterization

The genomic DNA from the isolates was prepared as described previously and used as template for PCR amplification [24]. The ITS region (ITS-1, 5.8 S rRNA and ITS-2) of rDNA was amplified with *Aspergillus* section *Flavi*-specific AFLF and AFLR primers as described in detail previously [10]. The species-specific identification of all isolates was studied by partial sequencing of β-tubulin and calmodulin gene fragments. The variable region of β-tubulin gene was amplified by using BTUBF (5^′^-TGGTAACCAAATCGGTGCTGCTT-3^′^) and BTUBR (5^′^-GCACCCTCAGTGTAGTGACCCT-3^′^) primers while the variable region of calmodulin gene was amplified by using Cmd5 (5^′^-GTCTCCGAGTACAAGGAGGC-3^′^) and Cmd6 (5^′^-TCGCCGATRGAGGTCATRACGTG-3^′^) primers and the amplicons were sequenced as described previously [25]. GenBank basic local alignment search tool (BLAST) searches (http://blast.ncbi.nlm.nih.gov/blast/Blast.cgi?) were performed for species identification. The DNA sequences for type strains already available in GenBank were retrieved. The gene sequences were analyzed individually or nucleotide sequences of both, β-tubulin and calmodulin gene fragments were included in the combined analysis. Multiple sequence alignments were performed with ClustalX version 2.0. The phylogenetic trees were constructed using the neighbor-joining method with pair-wise deletion of gaps option. *Aspergillus parasiticus* (CBS100926) was chosen as the outlying taxon and the robustness of branches was assessed by bootstrap analysis with 1,000 replicates.

### Antifungal susceptibility testing

#### Preparation of inoculum

All isolates were freshly sub-cultured on potato dextrose agar (PDA) slants to obtain good sporulation. The culture tubes were flooded with 1 ml of 0.9% saline and vortexed for 15 seconds to dislodge the conidia. The growth suspensions were transferred to another sterile tube containing 1.5-ml saline and 0.2% Tween 80. A conidial suspension containing approximately 0.4 × 10^4^ to 5 × 10^4^ cells was used as inoculum [13]. Reference strains of *A. flavus* (CBS100927) and *A. parasiticus* (CBS100926) were included with each batch of susceptibility testing to ensure quality control.

### Etest

Two media, namely, RPMI 1640 supplemented with 2% glucose (pH adjusted to 7.0 with morpholinepropanesulfonic acid buffer) and Mueller-Hinton (MH) II agar (Becton, Dickinson and Company, Sparks, MD, USA) supplemented with 2% glucose and methylene blue (0.5 μg/ml) (MH-GMB) were used for Etest. Etest was performed according to the manufacturer’s instructions Briefly, each 150-mm petri plate containing 60 ml of medium was inoculated by streaking the swab over the entire surface of the medium. Etest antifungal susceptibility strips for amphotericin B, voriconazole, posaconazole, caspofungin, anidulafungin and micafungin were obtained from bioMerieux Sa, Marcy-l’Etoile, France. Before applying Etest strips, the plates were allowed to dry for about 15 min. MIC readings were taken after 24 h of incubation at 35°C. The E-test MIC was defined as the lowest drug concentration at which the border of the elliptical inhibition zone intercepted the scale on the antifungal strip, Microcolonies within the inhibition zone were ignored. Considering that newer azoles are the treatment of choice for *Aspergillus* infections, the European Committee on Antifungal Susceptibility Testing (EUCAST): (http://www.eucast.org/antifungal_susceptibility_testing_afst/) has recently proposed the following clinical break-points for *A. fumigatus*: for itraconazole and voriconazole, ≤ 1 mg/L (susceptible) and >2 mg/L (resistant) and for posaconazole, <0.12 mg/L (susceptible) and >0.25 mg/L (resistant) [26]. For *A. flavus*, the clinical breakpoints for itraconazole are the same as for *A. fumigatus*, whereas for other antifungal agents there is insufficient evidence to recommend clinical breakpoints. The epidemiologic cutoff values proposed on the basis of MIC (mg/L) distribution of wild-type strains of *A. flavus* are as follows: itraconazole, 1 (99.6%); posaconazole, 0.25 (95%); voriconazole, 1 (98.1%); caspofungin, 0.5 (99%) and amphotericin B, 4 (99%), which are generally one step higher than *A. fumigatus*[14-16].

### Disk diffusion test

For the purpose of comparing zone of inhibition results, the disk diffusion test was performed on RPMI 1640 medium supplemented with 2% glucose instead of Mueller-Hinton agar medium. Disk diffusion disks for voriconazole (1-μg) and amphotericin B (100-μg) were obtained commercially (Bio-Rad Laboratories, Marnes-la-Coquette, France). After applying the discs, the plates were incubated at 35°C for 24 h. Zone diameters were measured at the point where the growth significantly decreased (80 to 100% inhibition) and were recorded to the nearest millimeter. Recently, Espinel-Ingroff et al. proposed for the first time quality control and reference zone diameter limits for *A. fumigatus* (ATCC MYA-3626) for amphotericin B (10-μg disk), itraconazole (10-μg disk), posaconazole (10-μg disk), and voriconazole (10-μg disk) [27]. No quality control ranges were recommended for *A. flavus* and *A. terreus*, because some results did not meet M23-A3 document requirements [28]. However, for *A. flavus*, the acceptable zone diameter ranges for voriconazole and posaconazole were proposed as 25–36 mm and 27–37 mm, respectively.

### Statistical analysis

The Spearman correlation test was performed to determine the correlation between Etest MICs and zone of inhibition diameters. A *P* value of <0.05 was considered as significant. Independent samples T test was used to compare mean MIC values of *A. flavus* strains isolated in two different periods of 9-year each (1993–2001 and 2002–2011).

## Results

### Molecular characterization

**Figure 1:**
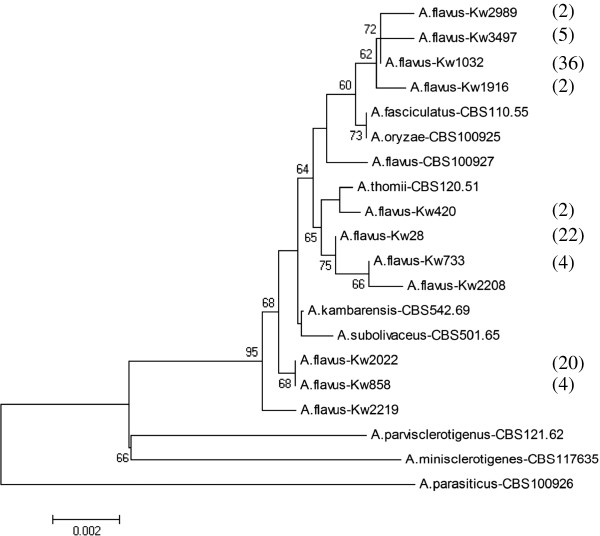
**Neighbor-joining phylogenetic tree based on combined β-tubulin and calmodulin gene sequence data for selected*A. flavus*isolates, each representing the 11 unique patterns, from Kuwait together with reference strains of several species belonging to*Aspergillus*section*Flavi*.** Total number of isolates in various clusters are indicated within brackets. Numbers on the nodes branches are bootstrap frequencies. Only values above 50% are indicated.

All 99 *A. flavus* isolates included in this study were identified as belonging to *Aspergillus* section *Flavi* by specific amplification of a DNA fragment of expected size (~243 bp) in PCR with *Aspergillus* section *Flavi*-specific primers AFLF and AFLR [10]. The partial β-tubulin DNA sequences from 94 isolates were completely identical and showed no difference with sequence from reference *A. flavus* (CBS100927) strain while the sequences of remaining 5 isolates differed at only 1 nucleotide position. The calmodulin gene sequences of the 99 isolates were more variable (total 8 patterns) but still showed maximum identity with sequence from reference *A. flavus* (CBS100927) strain than with reference strains of other species belonging to *Aspergillus* section *Flavi*. The β-tubulin and calmodulin gene sequences were combined and the combined data set was compared with corresponding combined data set from reference strains of *A. flavus* CBS100927, *A. parvisclerotigenus* CBS121.62, *A. minisclerotigenes* CBS117635, *A. parasiticus* CBS100926, *A. sojoe* CBS100928, *A. celatus* CBS763.97, *A. tamarii* CBS104.13 and *A. nomius* CBS260.88 strains. The combined sequences from *A. oryzae* CBS100925 and other synonyms of *A. flavus* (*A. fasciculatus* CBS110.55, *A. thomii* CBS120.51, *A. kambarensis* CBS542.69 and *A. subolivaceus* CBS501.65) were also used [29]. The combined β-tubulin and calmodulin gene sequences of the 99 isolates also showed maximum identity with sequence from reference *A. flavus* (CBS100927) strain. Pair-wise sequence comparisons showed that all 99 isolates exhibited 11 different patterns with three large clusters corresponding to 3 patterns containing 78 isolates (Table 2). The neighbor-joining phylogenetic tree based on combined data set for β-tubulin and calmodulin gene sequences (using only one representative isolate from each of 11 patterns) is shown in Figure 1. All 11 representative *A. flavus* isolates from Kuwait clustered together with *A. flavus* strains on a separate branch. The unique DNA sequencing data reported here have been submitted to EMBL under accession nos. HF570030-HF570051.

**Table 2:** **Nucleotide sequence differences in combined** β**-tubulin and calmodulin gene regions with the indicated sequence from reference*A. flavus*strain CBS100927**

**Pattern**	**No. of isolates**	**Representative isolate no.**	*β-tubulin region			*Calmodulin region									
			**332 C**	**390 G**	**57 Ins**	**98 G**	**121 G**	**132 T**	**154 Ins**	**166 T**	**177 T**	**197 A**	**452 G**	**485 C**	**563 C**
1^a^	36	Kw 1032			A					A		T			T
2^b^	22	Kw 28			A				T		C	T			T
3	20	Kw 2022			A				T			T	T		
4	5	Kw 3497			A	T				A		T			T
5	4	Kw 733			A			C	T		C	T			T
6	4	Kw 858			A				T			T	T		
7	2	Kw 2989			A					A		T		T	T
8	2	Kw 1916	A		A					A		T			T
9	2	Kw 420	A		A				T		C	T			T
10	1	Kw 2219			A		T		T		C	T	T		
11	1	Kw 2208		A	A			C	T		C	T			T

*The nucleotide positions are relative to the 5′-end of forward sequencing primers.

^a^Four environmental isolates belonged to this pattern.

^b^Three environmental isolates belonged to this pattern.

### Antifungal susceptibility

The results of Etest antifungal susceptibility of clinical isolates of *A. flavus* are presented in Table 3. In general, all tested antifungal agents showed good *in vitro* activity against *A. flavus*. The MIC range and MIC_90_ values on RPMI 1640 medium were as follows: amphotericin B, 0.064 – 4 and 3 μg/ml; voriconazole, 0.064 – 0.25 and 0.25 μg/ml; posaconazole, 0.016 – 0.38 and 0.25 μg/ml; anidulafungin, 0.002 – 0.006 and 0.002 μg/ml; micafungin, 0.002 – 0.008 and 0.002 μg/ml and caspofungin, 0.002 – 0.125 and 0.032 μg/ml, respectively. The MIC_90_ values obtained on MH-GMB were similar to RPMI values for all the tested antifungal agents except amphotericin B where it was 4 μg/ml.

**Table 3:** **Comparative minimum inhibitory concentration (MIC) values of 92 clinical isolates of*A. flavus*on RPMI 1640 and Mueller-Hinton agar media read at 24 hours**

**Antifungal drug**	**RPMI 1640**				**MH-GMB**			
	**Range**	**MIC 50**	**MIC 90**	**Mean ± SD**	**Range**	**MIC 50**	**MIC 90**	**Mean ± SD**
Amphotericin B	0.064-4	0.75	3	1.129 ± 0.982	0.1-6	1	4	1.646 ± 1.529
Voriconazole	0.064-0.25	0.125	0.25	0.153 ± 0.525	0.064-0.25	0.125	0.25	0.145 ± 0.047
Posaconazole	0.016-0.38	0.094	0.25	0.109 ± 0.075	0.023-0.5	0.094	0.25	0.142 ± 0.109
Anidulafungin	0.002-0.006	0.002	0.002	0.002 ± 0.000	0.002-0.016	0.002	0.002	0.00 ± 0.00
Micafungin	0.002-0.008	0.002	0.002	0.002 ± 0.002	0.002-0.008	0.002	0.002	0.002 ± 0.001
Caspofungin	0.002-0.125	0.008	0.032	0.016 ± 0.014	0.006-0.125	0.012	0.032	0.013 ± 0.015

SD- Standard deviation.

The cumulative percentage of *A. flavus* isolates inhibited at different MIC values are presented in Table 4. Of the 92 clinical isolates tested, 74.2% (n=69) and 59.1% (n=55) isolates were inhibited by amphotericin B at MIC of ≤1.024 μg/ml on RPMI and MH-GMB medium, respectively. About 11% (n=10) of the isolates showed MICs of >2 μg/ml on RPMI medium. Among azoles, voriconazole inhibited 100% of the isolates at a concentration of 0.256 μg/ml on both test media. At this concentration, posaconazole inhibited 100% of the isolates on RPMI and 93.5% of the isolates on MH-GMB. For three echinocandins, micafungin and anidulafungin demonstrated comparable *in vitro* activity inhibiting 100% of the isolates at a MIC of ≤0.008 μg/ml on RPMI medium, whereas caspofungin inhibited 63.4% of the isolates on RPMI and 33.3% of the isolates on MH-GMB. A comparison of the mean MIC values of *A. flavus* isolates recovered in two 9-year periods (1993–2001 and 2002–2011) did not show any significant difference for the antifungal drugs tested.

**Table 4:** **Distribution of*A. flavus*isolates according to MIC values**

		**Cumulative % of isolates inhibited at MIC of**												
**Antifungal agent**	**Medium**	**≤ 0.002**	**≤ 0.004**	**≤ 0.008**	**≤ 0.016**	**≤ 0.032**	**≤ 0.064**	**≤ 0.128**	**≤ 0.256**	**≤ 0.512**	**≤ 1.024**	**≤ 2.048**	**≤ 4.096**	**≤ 8.192**
Amphotericin B	RPMI						1.07	2.1	5.4	45.2	74.2	89.2	100	
	MH-GMB							3.2	4.3	36.6	59.1	81.7	96.8	100
Voriconazole	RPMI						3.2	67.7	100					
	MH-GMB						4.3	77.4	100					
Posaconazole	RPMI			1.07	3.2	17.2	47.3	78.5	100					
	MH-GMB			1.07	2.1	9.7	37.6	70.9	93.5	100				
Anidulafungin	RPMI	96.8	98.9	100										
	MH-GMB	89.2	93.5	98.9	100									
Micafungin	RPMI	95.7	96.8	100										
	MH-GMB	94.6	96.8	100										
Caspofungin	RPMI	5.4		63.4	87.1	97.8	98.9	100						
	MH-GMB	1.07	2.1	33.3	79.6	98.9		100						

The mean and range of MIC values of seven environmental *A. flavus* isolates on RPMI medium for the six antifungal agents were as follows: amphotericin B, 1.25 μg/ml ± 0.577 (range 0.75-2 μg/ml); voriconazole, 0.138 μg/ml ± 0.050 (range 0.094-0.25 μg/ml); posaconazole, 0.079 μg/ml ± 0.087 (range 0.008-0.25 μg/ml); anidulafungin, 0.002 μg/ml ± 0.00 (range 0.002-0.002 μg/ml); micafungin, 0.002 ± 0.00 (range 0.002-0.002 μg/ml) and caspofungin, 0.071 μg/ml ± 0.082 (range 0.008-0.19 μg/ml), respectively. These mean MIC values were comparable with those obtained on MH-GMB medium. None of the environmental isolates exhibited MIC value of >2 μg/ml for amphotericin B.

### Disk diffusion test

Using amphotericin B (100-μg) and voriconazole (1-μg) disks, the mean values for zone of inhibition for clinical isolates were 10.38 ± 1.655 mm (range 7–16 mm) and 28.88 ± 2.321 mm (24–34 mm), respectively (Table 5). For environmental isolates, the mean values for zone of inhibition were 10.71 ± 1.38 mm (range 8–12 mm) and 27.71 ± 1.603 mm (26–30 mm) for amphotericin B and voriconazole, respectively. The differences in inhibition zone diameters between clinical and environmental isolates for voriconazole and amphotericin B were not statistically significant. Linear regression analysis between Etest MIC values and disk diffusion diameters obtained with clinical isolates revealed a significant inverse correlation, both with amphotericin B (R^2^ = 0.2536, *p* <0.001) and voriconazole (R^2^ = 0.4043, *p*<0.003).

**Table 5:** Comparative results of disk diffusion tests for amphotericin B and voriconazole

**Antifungal drug**	**Clinical isolates (n=92)**				**Environmental isolates (n=7)**	
	**Range**	**Median**	**Mean ± SD**	**Range**	**Median**	**Mean ± SD**
Amphotericin B	7-16	10	10.38 ± 1.655	8-12	11	10.71 ± 1.380
Voriconazole	24-34	29	28.88 ± 2.321	26-30	27	27.71 ± 1.603

## Discussion

This study provides molecular characterization and *in vitro* antifungal susceptibility data on clinical and environmental isolates of *A. flavus* from Kuwait. All clinical and environmental isolates were identified as *A. flavus* strains based on the partial β-tubulin and calmodulin sequences. The phylogenetic tree derived from combined β-tubulin and calmodulin gene sequences also showed that all 99 isolates analyzed in this study clustered with reference strains of *A. flavus* or synonyms of *A. flavus*[29] clearly distinct from *A. parvisclerotigenus*, *A. minisclerotigenes* and *A. parasiticus,* the latter being used as an out-group. These studies established species-specific identity of all clinical and environmental isolates used in this study.

The *in vitro* drug susceptibility testing data showed that all the tested drugs exhibited good activity except amphotericin B, where ~10% isolates showed MIC of >2 μg/ml on RPMI medium. Although no antifungal susceptibility breakpoints are available for *A. flavus*, there is a consensus that isolates demonstrating MIC values of >1 μg/ml may be considered resistant to amphotericin B [26,30,31]. Badiee et al. [32] determined antifungal susceptibility of 66 *A. flavus* isolates from Iranian patients by Etest and CLSI methods. Thirty-six of the isolates were resistant to amphotericin B by both the methods. In another study from Tunesia, 31 of 37 isolates of *A. flavus* obtained from 14 patients with hematological malignancies were resistant (≥2 μg/ml) to amphotericin B [33]. Notably, survival rate was much lower among patients who yielded resistant strains (22% versus 67%) and were treated with amphotericn B. Similar results were obtained in a previous study [31]. However, with the introduction of voriconazole as a primary therapy for invasive aspergillosis, the concerns about amphotericin B resistance have been largely addressed in resource-rich countries.

All our *A. flavus* isolates were inhibited at a concentration of ≤0.256 μg/ml, which is lower or equal to the recently proposed clinical breakpoints for resistance to voriconazole (>2 μg/ml) and posaconazole (>0.25μg/ml) for *A. fumigatus* complex isolates [26]. Consistent with our observations, none of the 98 *A. flavus* clinical isolates tested in the Netherlands exhibited resistance to itraconazole or voriconazole [17]. So far, acquired resistance to azoles in *A. flavus* is extremely rare [34]. However, recent reports of voriconazole resistance in an *A. flavus* isolate cultured from lung specimen of a patient in China and a case of voriconazole-refractory eye infection in a patient from India have posed new therapeutic challenges [35,36]. It is unclear why itraconazole/voriconazole-resistance among *A. flavus* isolates is so rare despite their wide-spread environmental prevalence and exposure to same azole fungicides that are apparently related to development of resistance in *A. fumigatus*[17], more so, when voriconazole-resistant strains of *A. flavus* can be readily obtained in the laboratory [37]. Although we tested only seven environmental isolates of *A. flavus*, their mean MIC values were marginally lower than clinical isolates for voriconazole and posaconazole but higher for amphotericin B. In one previous study, MIC values of environmental isolates (n=59) for amphotericin B and itraconazole were found to be significantly lower than the clinical isolates (n=29) (*p* < 0.05) [38].

All three echinocandins showed good *in vitro* activity against *A. flavus* by Etest. Consistent with several previous reports, anidulafungin and micafungin appeared more potent than caspofungin [21,22,39,40]. Pfaller et al. reported that, caspofungin, micafungin and anidulafungin inhibited all *A. flavus* isolates (n=64) at a concentration of ≤0.06 μg/ml [39]. Recently, Espinel-Ingroff et al. determined epidemiological cut-off values (ECVs) for triazoles, caspofungin and amphotericin B using wild-type isolates of *Aspergillus* spp. employing CLSI microdilution methodology [14-16]. Our Etest MICs obtained with *A. flavus* isolates compared well with ECVs described in the above studies, involving 100% of the isolates with voriconazole, 98.9% of the isolates with posaconazole, and 100% of the isolates with caspofungin and amphotericin B (Table 6) [14-16]. Although we used Etest, the data suggest that there are no notable differences in the MICs of clinical and wild-type isolates of *A. flavus*. Furthermore, there was no significant difference in the mean MIC values of 14 *A. flavus* isolates recovered in the first 9-year period (1993–2001) compared to 78 *A. flavus* isolates recovered in the second 9-year period (2002–2011) for all the antifungal drugs tested.

**Table 6:** **Comparison of epidemiologic cutoff values (ECV) of*A. flavus*with Etest MICs obtained in the present study by Etest**

**Drug**	ECV (μg/μl)*	**% of isolates ≤ ECV**	
		RPMI 1640	MH-GMB
Voriconazole	1.0 (98.1%)	92 (100 %)	92 (100 %)
Posaconazole	0.25 (95 %)	91 (98.9 %)	86 (93.5 %)
Caspofungin	0.25 (95%)	92 (100 %)	92 (100%)
Amphotericin B	4.00 (99%)	92 (100%)	89 (96.7%)

*Espinel-Ingroff et al. [14-16].

Note: Epidemiologic cutoff of wild-type strains of *A. flavus* are not available for micafungin and anidulafungin.

A limitation of our study is the non-availability of BMD data for comparison with Etest MICs. Similar to some other studies, we also ignored the presence of microcolonies within the Etest inhibition zone [21,22]. A good concordance has been reported between MICs obtained by Etest and BMD test for posaconazole (84% to 98%) [41,42], itraconazole (100%) [43,44], and voriconazole (85%) [44-46] against *Aspergillus* spp. at both, 24 and 48 h readings in studies that have used these two methods. Recently, Colozza et al. compared CLSI and Etest MICs for determining *Aspergillus* spp. susceptibility to amphotericin B and AmBisome [30]. A high percentage of *A. flavus* complex isolates were resistant (>1 μg/ml) to AmBisome (43.7%) than amphotericin B (16.7%) by BMD. This was in contrast with the amphotericin B susceptibility results obtained by BMD method for the same isolates, but not with those obtained by the Etest. The authors inferred that Etest may be a superior method than BMD for determining amphotericin B-resistant *Aspergillus* strains, perhaps because of the wider range of MIC distribution available on the Etest strip.

There is paucity of data on correlation between inhibition zone diameters and Etest MICs against *Aspergillus* spp. A few studies available in the literature suggest that agar diffusion methods have potential value for evaluating susceptibility to antifungal agents [47,48]. In a multicenter study, Espinel-Ingroff et al. evaluated agar diffusion method for susceptibility testing of filamentous fungi on plain Muller-Hinton agar medium [47]. The investigators suggested that using 5-μg disk of posaconazole and caspofungin, 1-μg disk of voriconazole, and 10-μg disk for itraconazole, and an incubation time of 24 h are optimal for determining susceptibility of three *Aspergillus* spp. (*A. fumigatus, A. flavus,* and *A. niger*). Recently, Espinel-Ingroff et al. [27], following CLSI described guidelines [49], established zone diameter limits for disk diffusion susceptibility testing for *A. fumigatus*. No quality control ranges were recommended for *A. flavus* and *A. terreus* as some of the results did not satisfy the M23-A3 document requirements [28]. However, the zone diameter ranges of 27 to 37 mm for posaconazole and 25 to 36 mm for voriconazole obtained by the authors for *A. flavus* isolates were considered acceptable. We obtained a comparable zone diameter range of 24 to 34 mm with 1-μg voriconazol disk, whereas zone diameters for amphotericin B were much narrower (7 to 16 mm) despite the fact that we used 100-μg disks. More recently, Martos et al. evaluated disk diffusion method for determining susceptibility to echinocandins [21]. For all *Aspergillus* spp. (including 18 *A. flavus* isolates), caspofungin disk provided narrower zone of inhibition (14–29 mm) than micafungin (14–40 mm) and anidulafungin (22–45 mm) at 24 h incubation, which is in concordance with relatively higher Etest MICs obtained with caspofungin than micafungin and anidulafungin obtained in the present study.

## Conclusion

All 92 clinical isolates collected over an 18-year period in Kuwait and phenotypically identified as *A. flavus,* were also characterized as *A. flavus* strains by partial sequencing of β-tubulin and calmodulin gene fragments. The triazoles and echinocandins showed good activity against clinical as well as environmental *A. flavus* isolates, however, nearly 11% and 18% isolates showed MIC of >2 μg/ml against amphotericin B on RPMI agar medium and Mueller-Hinton agar medium, respectively. There was a significant inverse correlation between Etest MICs and inhibition zone diameter values with voriconazole and amphotericin B. To the best of our knowledge, this is the first study reporting results of molecular characterization of *A. flavus* isolates from the Middle East.

## Competing interests

The authors declare that they have no competing interests.

## Authors’ contributions

ZUK and SA conceived the study, supervised it and finalized the manuscript. FAW did the work which formed part of her Master thesis and contributed to writing of the manuscript. All authors have read and approved the final version of the manuscript.

## Pre-publication history

The pre-publication history for this paper can be accessed here:

http://www.biomedcentral.com/1471-2334/13/126/prepub
